# Comparative Efficacy of Dexmedetomidine and Remifentanil in Reducing Postoperative Pain and Opioid Use: A Systematic Review

**DOI:** 10.7759/cureus.79759

**Published:** 2025-02-27

**Authors:** Abbas Al-Hassan, Brandon Weissman, Shafayath Chowdhury, John Sawires, Varun Soti

**Affiliations:** 1 Anesthesiology and Critical Care, Lake Erie College of Osteopathic Medicine, Elmira, USA; 2 Internal Medicine, Lake Erie College of Osteopathic Medicine, Elmira, USA; 3 Pharmacology and Therapeutics, Lake Erie College of Osteopathic Medicine, Elmira, USA

**Keywords:** efficacy, opiate-free analgesia, opioid-induced hyperalgesia, remifentanil, dexmedetomidine

## Abstract

Postoperative pain management is a critical component of patient care following surgical procedures. Opioid analgesics, particularly Remifentanil, have been traditionally used to manage pain, but these drugs come with significant risks, including opioid-induced hyperalgesia (OIH) and the potential for dependence on opioids. Dexmedetomidine, an alpha-2 adrenergic receptor agonist, is a promising non-opioid alternative. This systematic review aims to compare the efficacy of Dexmedetomidine and Remifentanil in reducing postoperative pain and opioid use, as well as to evaluate their safety profiles. A systematic literature review followed the Preferred Reporting Items for Systematic Reviews and Meta-Analyses guidelines, utilizing PubMed and ClinicalTrials.gov. The findings revealed that both agents provided effective intraoperative analgesia. However, Dexmedetomidine consistently outperformed Remifentanil in reducing postoperative pain and opioid consumption. Additionally, Dexmedetomidine was associated with lower pain scores in the immediate postoperative period, particularly in spinal and gynecological surgeries. Despite its advantages in pain control and opioid-sparing effects, Dexmedetomidine was associated with slower recovery times, such as delayed eye-opening and longer extubation periods. In contrast, Remifentanil, with its rapid onset and offset, facilitated quicker recovery but resulted in increased postoperative opioid requirements and a higher incidence of OIH. Dexmedetomidine's side effects include bradycardia and hypotension, which were generally manageable and less severe than those associated with Remifentanil. Dexmedetomidine represents a promising alternative to Remifentanil for postoperative pain management. Its ability to reduce opioid consumption and mitigate the risk of OIH makes it an attractive option, particularly in patients at risk for opioid misuse or dependency. Nevertheless, further large-scale studies with diverse patient populations are needed to confirm these findings and refine the optimal dosing regimens for Dexmedetomidine.

## Introduction and background

Each year, millions of surgical procedures requiring general anesthesia, postoperative follow-up, and pain management are performed in the United States (U.S.). Among these, laparoscopic cholecystectomy, awake craniotomy, laparoscopic sleeve gastrectomy, gynecological laparoscopy, and major urologic procedures are widely conducted and necessitate effective intraoperative analgesia. Pain management in the postoperative period is a critical component of patient care, as inadequate pain control can lead to prolonged hospital stays, increased morbidity, and an elevated risk of chronic opioid use [1,2].

Opioid analgesics remain the cornerstone of postoperative pain management, with Remifentanil being one of the most commonly employed agents in this context. Opioids exert their analgesic effects by binding to opioid receptors, which are G-protein-coupled receptors (GPCRs) primarily found in the central and peripheral nervous systems. There are three main classes of opioid receptors: mu (μ), delta (δ), and kappa (κ). Among them, the mu-opioid receptor (MOR) is most critical for analgesia, as its activation leads to the inhibition of adenylyl cyclase, a reduction in 3,5-cyclic adenosine monophosphate (cAMP) levels, and decreased neurotransmitter release, thereby attenuating pain signals in the dorsal horn of the spinal cord [3,4].

Remifentanil, a potent, ultra-short-acting MOR agonist, provides rapid analgesia and sedation. However, chronic opioid exposure can lead to receptor desensitization, tolerance, and hyperalgesia due to compensatory upregulation of excitatory pathways. Prolonged opioid use is also associated with the downregulation of endogenous opioid production, predisposing patients to opioid withdrawal and dependence [5,6]. These effects contribute to the cycle of increased opioid requirements postoperatively, exacerbating the risk of opioid-induced hyperalgesia (OIH), prolonged hospital stays, and chronic opioid dependence [7,8].

The economic burden associated with opioid dependence is substantial. Studies have shown that opioid misuse results in increased hospitalizations, higher healthcare costs, and loss of productivity. The annual financial burden of opioid-related disorders in the U.S. healthcare system is estimated to be in the billions, with an increasing trend due to ongoing prescription opioid use in perioperative settings [9,10]. Furthermore, opioid use following surgery has been implicated in the development of chronic opioid dependence, with studies indicating that approximately 5.9-6.5% of surgical patients transition to chronic opioid use postoperatively [11]. Given that chronic opioid users have a 25% likelihood of developing opioid use disorder (OUD), the impact of opioid-based pain management on long-term patient outcomes cannot be understated [12,13].

An emerging alternative to opioid-based anesthesia is Dexmedetomidine, an alpha (α)-2 adrenergic receptor agonist with sedative, analgesic, and opioid-sparing properties. α2 adrenergic receptors are GPCRs located in the locus coeruleus and dorsal horn of the spinal cord. Activation of these receptors inhibits the release of norepinephrine, reducing sympathetic outflow and leading to enhanced analgesia [14,15]. Unlike Remifentanil, Dexmedetomidine does not induce respiratory depression or OIH, making it a promising candidate for opioid-free anesthesia [16].

The clinical application of α2 adrenergic receptor agonists for pain management dates back to the 1960s when clonidine was first introduced for its antihypertensive and sedative properties. Subsequent research demonstrated its analgesic potential, leading to the development of more selective agents such as Dexmedetomidine, which provides superior analgesia with fewer cardiovascular side effects [17,18]. Several studies have explored the intraoperative use of Dexmedetomidine in comparison to Remifentanil, evaluating its efficacy in reducing postoperative pain levels, minimizing the need for additional opioid analgesics, and mitigating the risk of developing OUD. In addition, Dexmedetomidine has demonstrated advantages in improving hemodynamic stability, reducing delirium, and enhancing postoperative recovery in various surgical populations [19,20].

This systematic review aimed to provide a comprehensive analysis of the existing literature regarding the efficacy of intraoperative Dexmedetomidine compared to Remifentanil. Specifically, assessed differences in postoperative pain outcomes, opioid consumption, OIH incidence, and the likelihood of long-term opioid use following surgery. By synthesizing current evidence, this paper attempted to aid and inform anesthesiologists and perioperative care providers on the potential benefits of incorporating Dexmedetomidine into clinical practice as an alternative to Remifentanil, ultimately contributing to improved patient outcomes and reduced opioid-related complications.

## Review

Method

**Figure 1:**
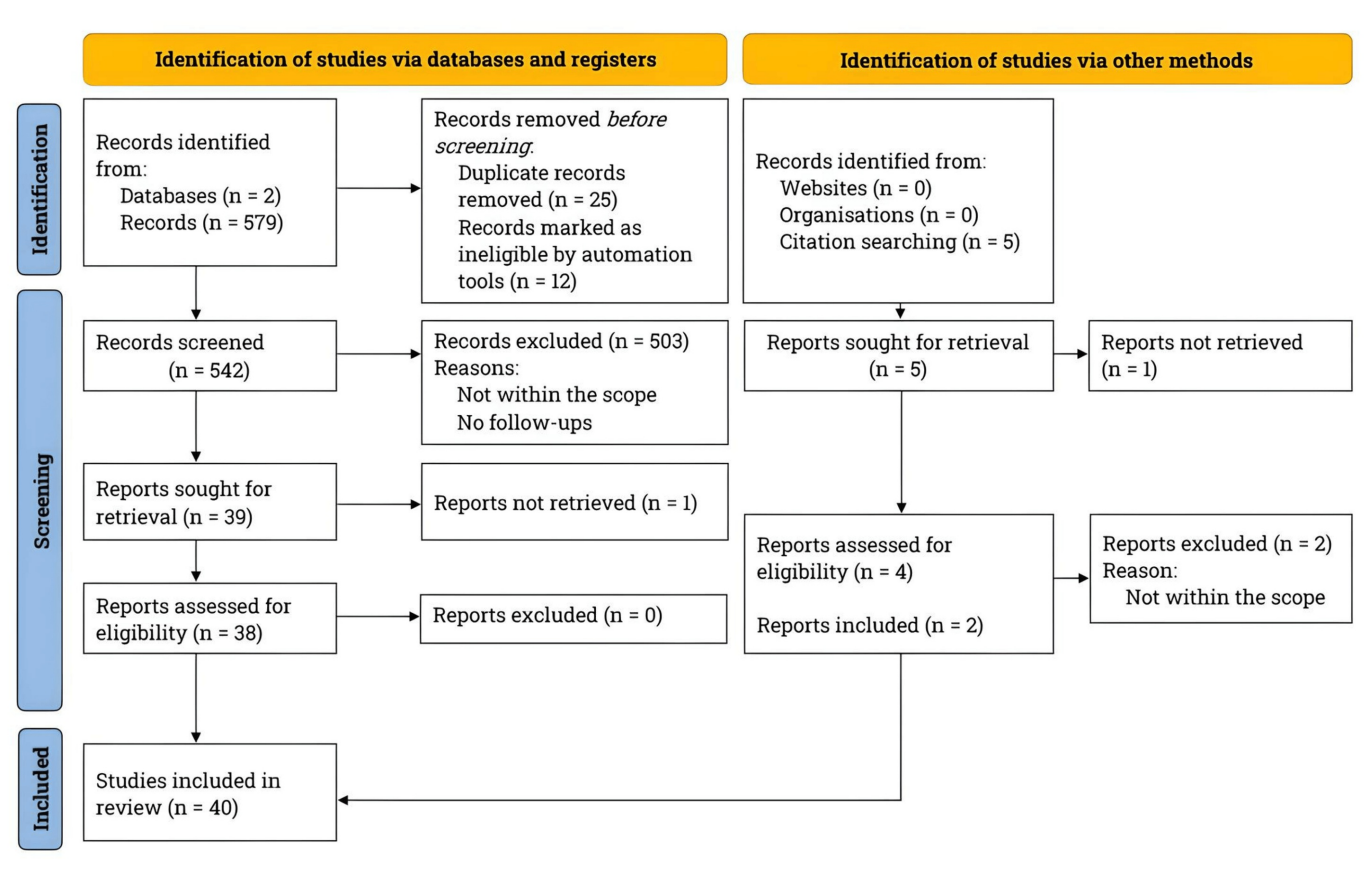
PRISMA flowchart for literature search and study selection n: number; PRISMA: Preferred Reporting Items for Systematic Reviews and Meta-Analyses

Following the Preferred Reporting Items for Systematic Reviews and Meta-Analyses (PRISMA) guidelines, we performed a literature search on PubMed and ClinicalTrials.gov from March 2023 to January 2025 [21]. Figure 1 illustrates the PRISMA flowchart detailing the literature search and study selection process.

The literature search utilized keywords such as “Remifentanil,” “Opioid analgesics in post-surgical management + Remifentanil,” “Opiate-free anesthesia,” “Dexmedetomidine,” “Opiate-free anesthesia + Dexmedetomidine,” and “Dexmedetomidine versus Remifentanil.”

Each included study was assigned a level of clinical evidence based on prior literature [22]. Table 1 summarizes the criteria for study inclusion.

**Table 1:** Study selection criteria The table delineates the inclusion criteria for selecting studies for this systematic review. We prioritized studies that directly compared the safety and efficacy of Dexmedetomidine and Remifentanil in the context of postoperative pain relief, as well as their effectiveness in reducing opioid-based pain management following surgery. Moreover, we directed our attention to studies investigating opioid receptors, the implications and adverse effects associated with the use of opioid analgesics in postoperative pain management, the function of alpha-2 adrenergic receptors in antinociceptive mechanisms, and research focused on Dexmedetomidine, including its pharmacokinetic and pharmacodynamic properties. Our careful methodology ensured that only the most relevant and credible studies published in English, which fulfilled the specified criteria in the table, were accorded higher priority.

Inclusion criteria for studies that directly compared the safety and efficacy of Dexmedetomidine and Remifentanil	Inclusion criteria for studies on opioid receptors, opioid analgesics in post-surgical pain management, the role of alpha-2 adrenergic receptors in antinociception, Dexmedetomidine approval, pharmacokinetics, and pharmacodynamics
Randomized controlled trials	Meta-analyses
Non-randomized controlled trials	Systematic reviews
Prospective clinical studies	Narrative reviews
Observational studies	Commentaries
Comparative studies	Opinions
Pilot studies	Pre-clinical studies
Case series	Randomized controlled trials, non-randomized controlled trials, prospective clinical studies, observational studies, comparative studies, pilot studies, case series, and case reports
Case reports	

Table 2 presents the categorized breakdown of studies in this review.

**Table 2:** Total number of studies reviewed The table displays the breakdown of studies included in this systematic review, organized by category.

Category	Number of studies included
Pain management in the postoperative period	2
Physiology of opioid receptors in pain control	6
Economic and public health burden of opioid use	5
Role of alpha-2 adrenergic receptors in pain modulation	3
Remifentanil: pharmacokinetics and pharmacodynamics	5
Dexmedetomidine: pharmacokinetics and pharmacodynamics	5
Method for systematic literature search	1
Assignment of level of clinical evidence to selected studies	1
Dexmedetomidine as an alternative to Remifentanil	2
Clinical evidence of direct comparison of the safety and efficacy of Dexmedetomidine and Remifentanil	10
	Total studies = 40

Results

Remifentanil Use Intraoperatively

Remifentanil was initially approved for clinical use in 1996 [23]. It has since become a standard agent in the practice and administration of anesthesia, utilized in approximately 34% of general anesthesia cases due to its ability to reduce pharmacokinetic variability and dosage discrepancies among patients. The medication acts rapidly, exhibiting a half-life of 10 to 20 minutes. During surgical procedures, Remifentanil is frequently administered as a continuous intravenous infusion, facilitating the achievement of adequate analgesia while optimizing recovery time and minimizing hemodynamic fluctuations. As a μ-opioid agonist, Remifentanil is rapidly metabolized through the hydrolysis of the propanoic acid-methyl ester linkage by nonspecific blood and tissue esterases, which accounts for its short duration of action [24].

Remifentanil

Remifentanil is recognized as a well-established, rapidly acting analgesic. However, it has been shown to contribute to OIH, a phenomenon where nociceptors become sensitized due to opioid exposure, including Remifentanil. This sensitization can result in heightened postoperative pain sensations for patients. As a consequence, OIH can lead to increased opioid consumption and elevated pain levels following surgery. Research indicates that Remifentanil is associated with OIH at a rate of 16.1% [25]. This finding underscores the significant relationship between Remifentanil administration and the challenges of postoperative pain management. Given that opioids are the primary approach for postoperative pain relief, there exists a direct correlation between remifentanil use and the rising rates of postoperative opioid requirements [26-27].

Dexmedetomidine: A Non-opiate Analgesic

Approved by the Food and Drug Administration (FDA) in December 1999, Dexmedetomidine is an α2-adrenoceptor agonist introduced for clinical practice as a short-term sedative for procedures lasting under 24 hours. Its potency indicates that complete anesthesia can be achieved, particularly compared to weaker α2-adrenoceptor agonists like Clonidine. The presynaptic α2-receptors targeted by Dexmedetomidine are clinically significant due to their negative feedback mechanism, which regulates the release of norepinephrine and adenosine triphosphate by activating regulatory G proteins. This mechanism underlies the inhibition of pain signal propagation [28].

The activated second messenger system inhibits adenylate cyclase, decreasing cAMP formation. This process facilitates the efflux of potassium through activated channels, hyperpolarizing the membrane potential, suppressing neuronal firing, and stimulating α2-adrenoceptors that inhibit calcium entry into the nerve terminal. Furthermore, post-synaptic activation of α2-adrenoceptors within the central nervous system (CNS) reduces sympathetic activity, thereby decreasing blood pressure and heart rate [28].

This neuronal hyperpolarization is the basis for the action of α2-adrenoceptor agonists such as Dexmedetomidine, producing analgesia, sedation, and anxiolysis while minimizing the side effects associated with multi-agent therapies. This mechanism allows for the achievement of an impact similar to those made by Remifentanil while circumventing the stimulation of opiate receptors, thus reducing the risk for OIH [28].

When Dexmedetomidine is utilized as an alternative to Remifentanil during surgical procedures, research has demonstrated a notable reduction in the need for postoperative pain management. A 2022 study involving 257 patients revealed that, within the first 24 hours after surgery, 87% of patients in the opiate-free anesthesia group did not require morphine, in contrast to 52% of those in the opiate-based group receiving Remifentanil. This difference remained significant even after adjusting for age, body mass index, sex, type of surgery, and procedure duration. Additionally, Dexmedetomidine was linked to a reduced requirement for postoperative morphine compared to Clonidine, a relatively weaker α2-adrenoceptor agonist, a finding that aligns with Dexmedetomidine's greater potency [29].

It is plausible that the longer duration of action exhibited by Dexmedetomidine, compared to Remifentanil, contributes to the lower levels of postoperative pain observed. The ability of Dexmedetomidine to effectively manage pain while minimizing pharmacological interaction with opiate receptors both during and after surgery suggests a potential decrease in the risk of patients developing chronic opiate dependence, thereby reducing overall exposure to opiates [30].

Effectiveness of Dexmedetomidine Compared to Remifentanil

Over the years, researchers have investigated the levels of postoperative opiate use of Dexmedetomidine and Remifentanil. A notable study by Hwang et al. (2015) explored postoperative pain levels following spinal surgery through a randomized controlled trial between 2013 and 2014. The researchers enrolled 40 subjects aged 18 to 70 years who met the American Society of Anesthesiologists (ASA) physical status I or II criteria. They were undergoing surgery for lumbar herniated nucleus pulposus, spinal stenosis, spondylolysis, spondylolisthesis, and posterior lumbar interbody fusion. Of the initial 40 participants, 37 were included in the final results, with 18 in the remifentanil group and 19 in the Dexmedetomidine group. Patients did not receive premedication before induction. In the Remifentanil group, a 0.01 microgram/kilogram/minute (μg/kg/min) was administered through target-controlled infusion, discontinued upon skin closure completion. In contrast, the Dexmedetomidine group received a continuous infusion of 0.01 μg/kg/min using a syringe pump, which was also halted at the start of skin closure. During their stay in the post-anesthesia care unit (PACU) and the ward, patients were given 1 μg/kg of Fentanyl and 50 milligrams (mg) of Tramadol as rescue analgesics [31].

Post-surgery, various metrics were recorded, including the visual analog scale (VAS) score, amount of patient-controlled analgesia (PCA) used, rescue analgesics required, and incidences of postoperative nausea and vomiting (PONV). Data was collected at discharge from the PACU (T1) and subsequently at 2 (T2), 8 (T3), 24 (T4), and 48 hours (T5) post-operation. The findings indicated a significant delay in eye-opening time (p = 0.001) and first verbal command response (p = 0.027) for the Dexmedetomidine group. At each measured interval (T1, T2, T3, T4, and T5), those in the Dexmedetomidine group required significantly fewer rescue analgesics (p = 0.018, p = 0.046, p = 0.038, p = 0.045, and p = 0.038, respectively). Furthermore, the incidence of PONV was notably lower at T1, T2, T3, and T4, with no significant difference observed at T5 (p = 0.003, p = 0.008, p = 0.029, p = 0.008, and p = 0.230, respectively). The VAS scores and PCA usage in the Dexmedetomidine group were persistently lower than in the Remifentanil group across all postoperative time intervals (p < 0.05) [31].

These results suggest that patients in the Remifentanil group experienced higher pain levels at all recorded time points and necessitated greater opiate-based rescue analgesics (Tramadol and Fentanyl). In contrast, those receiving Dexmedetomidine required fewer opiate interventions over the 48 hours following surgery. This finding has important implications, as reducing the number of opiate prescriptions post-surgery may decrease the likelihood of patients developing chronic opiate use. However, the significance of these results may be tempered by the relatively small sample size (n = 37) [31].

Kim et al. (2021) conducted a prospective study to compare the recovery profiles associated with the perioperative use of Dexmedetomidine versus Remifentanil in patients undergoing laryngeal microsurgery (LMS). One of the primary assessments was postoperative opioid use, which was quantified by the amount of rescue Fentanyl administered in the PACU. A total of 61 LMS patients were randomly allocated to either the Dexmedetomidine infusion group or the Remifentanil group. The demographic and clinical characteristics, including age, sex, height, weight, body mass index (BMI), presence of hypertension, and preoperative hemoglobin levels, did not show significant differences between the two groups [32].

The study protocol involved a continuous intravenous infusion of the assigned study drug starting 10 minutes before anesthesia induction and continuing until the end of the surgical procedure. In the Dexmedetomidine group, a loading dose of 1.0 µg/kg was prepared by diluting Dexmedetomidine with saline. Anesthesia induction was achieved using 1.5 mg/kg Propofol and 0.5 mg/kg Rocuronium, followed by a maintenance infusion of 0.005 to 0.01 µg/kg/min during the surgery. For the Remifentanil group, saline served as a placebo loading dose during the first 10 minutes at the same flow rate as administered to the Dexmedetomidine group. One minute before induction, 1.0 µg/kg of Remifentanil diluted with saline was given, followed by the same induction protocol using Propofol and Rocuronium as the Dexmedetomidine group. The maintenance dose for Remifentanil was set at 0.05 to 0.1 µg/kg/min [32].

The findings indicated that postoperative pain scores in the PACU were significantly higher in the Remifentanil group (p = 0.032). Moreover, the average Fentanyl consumption per patient was markedly lower in the Dexmedetomidine group compared to the Remifentanil group (3.3 µg vs. 23.2 µg, respectively; p < 0.001). However, the time to eye-opening was notably longer in the Dexmedetomidine group, averaging 599.4 seconds, compared to 493.6 seconds in the Remifentanil group (p < 0.01). This study illustrates that the use of Dexmedetomidine instead of Remifentanil can lead to significantly reduced pain levels and a substantial decrease in opioid consumption per patient [32].

In a recent study by Koo et al. (2023), the postoperative side effects of opiates were examined with a focus on comparing the efficacy of Dexmedetomidine and Remifentanil in mitigating opioid-related complications during gynecological laparoscopy. This prospective, parallel-group, single-blind, randomized controlled trial involved 96 female participants, aged 20-65 years, undergoing various procedures such as hysterectomy, oophorectomy, salpingectomy, cystectomy, cyst enucleation, and uterine myomectomy. Patients were randomly assigned to the Remifentanil or Dexmedetomidine group [33].

Participants in the Dexmedetomidine group received an initial loading dose of 0.7 μg/kg for 10 minutes before anesthesia induction, followed by a continuous infusion of 0.5 μg/kg/hour (h), with adjustments made in increments of 0.1 μg/kg/h to maintain systolic blood pressure within ±20% of baseline; the infusion was discontinued after surgery. Conversely, those in the Remifentanil group were administered a loading dose of 1.5 μg/kg, with a continuous infusion rate of 0.15 μg/kg/min, also adjusted by 0.02 μg/kg/min for blood pressure maintenance, and was stopped post-surgery. The hemodynamic impacts of both medications were evaluated, focusing on the incidence of intraoperative bradycardia (heart rate <60/min) and hypotension (systolic blood pressure <80 millimeters of mercury (mmHg) or mean arterial pressure (MAP) <60 mmHg). Recovery metrics such as time to eye-opening and extubation were documented [33].

Pain levels were quantified using a 0-10 numerical rating scale (NRS) 30 minutes post-surgery, upon arrival in the recovery room, and every 15 minutes thereafter. The duration of recovery and sedation levels was monitored at 30-minute intervals post-operation. The study also tracked instances of PONV, shivering, and any additional needs for analgesics or antiemetics, with a 24-hour follow-up post-surgery. Furthermore, oxidative stress was assessed through a cytokine study involving 20 patients from each group, measuring levels of manganese superoxide dismutase and matrix metalloproteinase-9 at baseline immediately after surgery and 24 hours thereafter [33].

The intraoperative analysis revealed no significant differences in anesthetic duration, time to eye-opening, time to extubation, or incidence of intraoperative bradycardia between the two groups. However, there was a notable disparity in the rates of intraoperative hypotension, with Remifentanil exhibiting a 45.8% incidence compared to 22.9% with Dexmedetomidine (p = 0.018). Postoperatively, pain scores recorded 30 minutes after recovery indicated that patients in the Dexmedetomidine group experienced significantly lower pain levels (NRS 4.0 ± 1.9) compared to those in the Remifentanil group (NRS 6.1 ± 2.0, p < 0.001). Additionally, a higher proportion of patients in the Dexmedetomidine group did not require analgesics during recovery (p = 0.002), although no significant differences in postoperative pain levels were observed between groups at the 24-hour mark [33].

In a study conducted by Hamed et al. (2019), the effects of Dexmedetomidine as an adjuvant to Sevoflurane anesthesia were evaluated in postoperative analgesia. This investigation utilized a prospective randomized clinical trial design, comparing intraoperative Dexmedetomidine to Remifentanil infusions during Sevoflurane anesthesia, specifically for gastric sleeve surgery. The study included 132 participants classified as ASA physical status II or III, all undergoing bariatric surgery with a BMI exceeding 35. Participants were equally divided, with 66 assigned to the Dexmedetomidine group and 66 to the Remifentanil group [34].

Preoperatively, all patients received anti-thrombotic prophylaxis and 40 mg of Pantoprazole intravenously. Patients were preoxygenated to maintain a peripheral oxygen saturation (SpO_2_) range of 97-99%. Additionally, all patients were given 1 mg of Droperidol and 0.25 mg/kg of Dexamethasone as prophylaxis against PONV. Before anesthesia induction, patients were administered Midazolam at 0.05 mg/kg. Anesthesia induction comprised 2 mg/kg of Propofol, 1-2 μg/kg of Fentanyl, and 0.15 mg/kg of Cisatracurium. After intubation, the lungs were ventilated with 100% oxygen using a semi-closed circle system for a tidal volume of 6-8 mL/kg, with the ventilatory rate adjusted to maintain an end-tidal carbon dioxide level of 32-35 mmHg. Balanced anesthesia was achieved with 1.24% end-tidal Sevoflurane in oxygen and air. Patients in the Remifentanil group received Remifentanil at rates of 6-18 μg/kg/h, while those in the Dexmedetomidine group received Dexmedetomidine at rates of 0.2-0.5 μg/kg/hr. Infusions for both groups commenced post-intubation and concluded upon completing the gastric sleeve procedure and removing the stomach via the trocar. Postoperatively, neuromuscular blockade was reversed with an intravenous injection of 0.05 mg/kg of Neostigmine and 0.02 mg/kg of intravenous Atropine. All patients were administered 20 mg of Pethidine, with an additional 50 mg available for rescue analgesia every four hours as needed [34].

Data collection encompassed various intraoperative metrics, including heart rate and MAP at specific intervals: T1 (before induction), T2 (after induction), T3 (five minutes post-insufflation), T4 (five minutes post-insufflation), and T5 (post-extubation). Furthermore, parameters such as duration of surgery, occurrences of intraoperative anesthetic or surgical problems, and emergence times were recorded. Postoperative data included MAP, heart rate, oxygen saturation, and severity of postoperative shoulder tip pain assessed at 1, 3, 6, 12, and 24 hours after transfer to the PACU, using an 11-point numeric scale. Postoperative wound pain was evaluated with a four-point verbal analog score. The study also recorded occurrences of PONV, frequency of antiemetic therapy (specifically, Ondansetron 4 mg intravenous), instances of postoperative complications, intensive care unit (ICU) admissions, and duration of hospital stay [34].

The findings indicated no significant difference in mean heart rate between the two groups at T1, T2, T3, and T5; however, at T4, the Remifentanil group displayed a significantly lower mean heart rate (p = 0.005). Sevoflurane consumption was notably less in the Dexmedetomidine group (p = 0.006). Emergence time until awakening, time until readiness for PACU transfer, and total operating room time were significantly shorter for the Remifentanil group than the Dexmedetomidine group (p = 0.001, p < 0.001, and p = 0.003, respectively). The mean duration of postoperative analgesia was also considerably shorter in the Remifentanil group (p < 0.001). Pain levels indicated that postoperative shoulder tip pain was significantly higher in the Remifentanil group at the three-hour mark (p < 0.05), while requests for rescue analgesia were substantially lower in the Dexmedetomidine group (p = 0.016). Additionally, postoperative wound pain at rest was significantly less in the Dexmedetomidine group compared to the Remifentanil group at multiple time points (p < 0.001, p < 0.001, p < 0.001, p = 0.004, p = 0.002 for 1 hour, 3 hours, 6 hours, 12 hours, and 24 hours, respectively). At the 48-hour postoperative mark, wound pain showed no significant difference between the groups. Wound pain during mobilization was also significantly less in the Dexmedetomidine group at various time points (p < 0.001 across 1 hour, 3 hours, 6 hours, and 12 hours), with no significant difference at 48 hours [34].

The results confirm a significant decrease in pain levels up to the 24-hour mark, while no differences were noted at the 48-hour point. This supports the speculation that Dexmedetomidine contributes to reduced pain following surgery, potentially leading to decreased opioid consumption and a lower risk of addiction. However, it is essential to note that the sample size in this study was relatively small [34].

Goettel et al. (2016) compared the efficacy and safety of Dexmedetomidine versus Propofol and Remifentanil-based conscious sedation regimens in awake craniotomies (AC) aimed at supratentorial tumor resection. Remifentanil, in this context, poses risks due to the unsecured airways of AC patients. Previous studies have highlighted that Propofol and Remifentanil can be associated with intraoperative airway and respiratory complications and reduced patient cooperation during cortical mapping [35].

The study included 50 adult patients aged over 18 who were undergoing anesthesia care. These patients were randomly assigned to two groups: one receiving Dexmedetomidine and the other receiving a combination of Propofol and Remifentanil, with 25 patients in each group. The primary outcome focused on the feasibility of intraoperative brain mapping, measured using a NRS. The secondary outcome evaluated the effectiveness of sedation through the modified Observer's Assessment of Alertness/Sedation (OAA/S) scale. Additional assessments included hemodynamic and respiratory variables, pain, sedation, anxiety scores, adverse events, and patient satisfaction. Importantly, no statistically significant differences were found in patient demographics - such as age, weight, height, gender, preoperative ASA physical status, medical comorbidities, or duration of anesthesia - between the two groups [35].

Dexmedetomidine was administered with a loading dose of 1 µg/kg over 10 minutes, followed by a maintenance infusion titrated to effect, with dosages ranging from 0.2 to 1 μg/kg/min. The Propofol and Remifentanil infusion rates were 25-150 µg/kg/min and 0.01-0.1 µg/kg/min, respectively. The dosing of all medications was adjusted to achieve a targeted sedation level of 2-4 points on the modified OAA/S scale. After establishing peripheral venous access in the operating room, each participant received 50 µg of Fentanyl, followed by the respective study drug infusions according to the sedation protocol. In cases of anticipated excessive pain or patient complaints of discomfort, the infusion rates of the study drug were increased, and rescue medication in the form of a Propofol bolus (20-30 mg, intravenous) was employed when first-line treatment was inadequate [35].

The intraoperative brain mapping proved successful in all patients, yielding an overall mean NRS score of 9.84 (range: 8-10). There was no significant difference in the ability to perform brain mapping between the two groups, with NRS scores showing Dexmedetomidine at 10.0 (95% confidence interval: 9.9-10.0) and Propofol/Remifentanil at 9.7 (95% confidence interval: 9.5-10.0), resulting in a p-value greater than 0.13. The level of sedation did not significantly differ between the groups at the time of mapping (p = 0.51). Furthermore, pain scores were significantly lower in the Dexmedetomidine group during skin incision and brain mapping (p = 0.026 and p = 0.031, respectively), although there were no differences noted at other stages of the procedure (p > 0.05) [35].

MAP was significantly lower in the Dexmedetomidine group during the dura opening, brain mapping, the start of tumor resection, upon admission to the PACU, and 120 minutes after admission to the PACU (p = 0.026, p = 0.007, p = 0.022, p < 0.001, and p = 0.004, respectively). At the same time, no significant differences were observed at other time points. Over time, heart rate also showed a substantial decrease in the Dexmedetomidine group, with a mean difference of -13.8 (-19.3, -8.4) beats per minute (p < 0.001). Although respiratory rates were lower in the Propofol and Remifentanil groups during the start of tumor resection and at skin closure (p = 0.030 and p = 0.002, respectively), both groups maintained typical respiratory rates (12-20 breaths per minute) at all measured time points [35].

The findings from this study suggest that there were either no differences or more favorable outcomes associated with the Dexmedetomidine group compared to the Propofol and Remifentanil group. This evidence further supports the notion that Dexmedetomidine could be a preferable alternative to the standard use of Remifentanil, even when combined with Propofol. The lower pain scores observed throughout the procedure potentially indicate a reduced requirement for postoperative pain medications, facilitating a diminished opioid profile. Such a reduction in Remifentanil usage may lower the risk of OIH while also decreasing the likelihood of the development of OUD, thus contributing to improved postoperative pain management [35].

Bakan et al. (2015) conducted a prospective, randomized, double-blind study to examine postoperative opioid consumption following laparoscopic cholecystectomy. The study involved 197 subjects aged 20-60 who met ASA physical status I or II and were scheduled for elective laparoscopic cholecystectomy. Ultimately, 85 subjects were included in the analysis, with 42 assigned to the opioid-based group (RF) and 43 to the opiate-free group (DL) [36].

In the DL group, participants received an initial loading dose of Dexmedetomidine at 0.6 μg/kg, followed by a continuous infusion of 0.3 μg/kg/h, alongside Lidocaine administered as a loading dose of 1.5 mg/kg and a maintenance infusion of 2 mg/kg/h. In contrast, the RF group was treated with Fentanyl and Remifentanil at a continuous rate of 0.25 μg/kg/min. Infusions for both groups were discontinued during the skin closure phase. The researchers collected a variety of data, including patient demographics, smoking history, instances of motion sickness and PONV, surgery duration, drug utilization during surgery, postoperative Fentanyl consumption over six hours, NRS scores, the incidence of PONV, and other adverse events. Additionally, a PCA system was established in the postoperative environment, delivering Fentanyl with a bolus of 20 μg and a lockout interval of five minutes [36].

The study findings revealed that the DL group exhibited a statistically significant reduction in cumulative postoperative Fentanyl use during the first two hours after surgery (p = 0.04). However, there was no statistically significant difference in cumulative Fentanyl consumption in the 0-4 or 0-6 hour postoperative periods. Furthermore, the DL group reported a significantly lower need for rescue analgesics (p = 0.034). Notably, the DL group also demonstrated elevated heart rates and MAPs at intubation and at the 1st, 4th, 7th, and 10th minutes following pneumoperitoneum (p < 0.05). Importantly, Ondansetron was not needed for PONV in the DL group, unlike in the RF group (p = 0.026) [36].

Additionally, patients in the RF group required considerably more Ephedrine to manage hypotension (p = 0.029). These results underscore the distinct differences in opioid consumption levels between the two groups within the first two hours postoperatively, highlighting a reduction in prescribed opioids. The study further illustrates a significant decrease in the requirement for rescue analgesics, which are typically opioid-based. While the sample size was small, these findings strongly advocate for the reduced need for opioids when using Dexmedetomidine compared to Remifentanil in the postoperative setting [36].

Pan et al. (2019) conducted a randomized control trial to evaluate the potential of Dexmedetomidine as a substitute for Remifentanil during ultrasound-guided radiofrequency ablation (RFA) of small hepatocellular carcinoma (HCC). The study primarily focused on comparing postoperative pain intensity, while secondary objectives included assessing analgesic requirements, postoperative liver function, patient comfort, and hemodynamic changes. Ninety-four patients were randomly assigned to two groups of 47 undergoing RFA for HCC; one group received Dexmedetomidine, while the other was administered Remifentanil. Key demographics and baseline laboratory values - such as age, sex, body mass index, alanine aminotransferase (ALT), aspartate aminotransferase (AST), albumin, total bilirubin, and prothrombin time - showed no significant differences between the groups [37].

In this investigation, patients were not premedicated. The Dexmedetomidine group received a bolus infusion of 200 μg, diluted to 4 μg/mL, at a rate of 0.4 μg/kg, 15 minutes before anesthesia induction, followed by a continuous infusion of 0.2 μg/k/h until 10 minutes before the surgery concluded. Conversely, the Remifentanil group was administered 1 mg of Remifentanil, diluted to 20 μg/mL, continuously at 3 μg/kg/h starting 15 minutes before induction and continuing until the end of the procedure. Propofol (1.5 mg/kg), Fentanyl (3.0 μg/kg), and Cisatracurium (0.2 mg/kg) were utilized for induction [37].

Findings revealed no significant differences between the two groups in VAS or postoperative pain levels. However, both groups postoperatively exhibited a notable reduction in liver function tests (AST, ALT, albumin, and prothrombin levels). Heart rates were significantly lower in the Dexmedetomidine group compared to the Remifentanil group at one minute after intubation (70.62 ± 12.93 vs. 75.38 ± 15.46, p = 0.018), 30 minutes into surgery (59.21 ± 8.26 vs. 67.76 ± 12.90, p < 0.001), and during recovery (p < 0.005). Notably, there were no significant differences in the incidence of hypotension or bradycardia between the groups. For all measured parameters, sevoflurane concentration was significantly lower in the Dexmedetomidine group (p < 0.005), with a marked reduction in total sevoflurane dosage (22.77 ± 11.18 vs. 17.58 ± 11.22 mL, p = 0.017). No significant differences were observed in delayed emergence, time to emergence, or extubation time [37].

The study concludes that despite no difference in postoperative pain levels, the lower Sevoflurane requirements in the Dexmedetomidine group may help mitigate associated side effects. Anesthesia delivery remained consistent across both groups, and patients experienced similar emergence times, even when substituting an opioid with Dexmedetomidine [37].

Janatmakan et al. (2021) examined the comparative efficacy of Remifentanil versus Dexmedetomidine for pain control in spinal surgery patients. This double-blind, randomized clinical trial aimed to evaluate pain management outcomes for 60 patients undergoing lumbar discectomy. The patients were divided into two groups, and they received either Dexmedetomidine or Remifentanil. No significant differences in sex, age, ASA class, or other demographic data were found between the groups [38].

Both groups received identical medications in the operating room, which included 5 mL/kg of crystalloid fluid, 2 μg/kg of Fentanyl, and 0.02 mg/kg of Midazolam as premedications. Anesthesia induction utilized 1.5 mg/kg and 0.5 mg/kg of Atracurium. The Dexmedetomidine group was administered 1 μg/kg of Dexmedetomidine over 10 minutes, followed by a continuous infusion of Propofol at 100-150 μg/kg/min along with a continuous Dexmedetomidine infusion of 0.01 μg/kg/min. In contrast, the Remifentanil group received a 1 μg/kg bolus with continuous Propofol at 150-100 μg/kg/min and Remifentanil infusion at 0.1 μg/kg/min [38].

The results indicated that the mean pain intensity in the Dexmedetomidine group was significantly lower than that in the remifentanil group (Remifentanil: 3.80 ± 1.1, Dexmedetomidine: 2.98 ± 1.29, p < 0.001). Although no significant differences in heart rate, MAP, or SpO_2_ were noted before surgery, during the procedure, and up to 120 minutes PACU, both mean heart rate and MAP were significantly lower in the Dexmedetomidine group (p < 0.05). Additionally, the frequency of nausea and vomiting was reduced considerably immediately upon entering the PACU and at 30 minutes and 120 minutes post-surgery (p < 0.05). At other time intervals assessed, nausea and vomiting frequencies showed no significant differences between groups. Ultimately, this study highlights the effectiveness of Dexmedetomidine in reducing postoperative pain, as well as the decreased incidence of nausea and vomiting [38].

Rahimzadeh et al. (2015) compared the effectiveness of Remifentanil and Dexmedetomidine in managing hemodynamics for patients undergoing posterior spinal fusion surgery. This double-blind, randomized clinical trial involved 60 patients between 15 and 65. Key parameters measured included intraoperative blood pressure, heart rate, mean sedation scores post-extubation, and pain scores in the recovery phase, which were crucial for evaluating the efficacy of the two agents [39].

The patients were randomly divided into groups of 30. One group received Remifentanil intraoperatively, while the other was administered Dexmedetomidine. Anesthesia was induced in all participants using Fentanyl at 3 µg/kg and Midazolam at 0.1 mg/kg for premedication. This was followed by administering Cisatracurium at 0.2 mg/kg and Sodium thiopental at 5 mg/kg. For maintenance during surgery, both groups were administered Isoflurane at 1%. Notably, Isoflurane was selected over the commonly utilized Propofol, as Propofol can decrease heart rate and induce bradycardia, potentially influencing the data due to similar side effects of Dexmedetomidine. Isoflurane was deemed a more appropriate inhalational anesthetic agent for this study [39].

In the Remifentanil group, a 1 µg/kg loading dose was infused over 15 minutes, followed by a continuous infusion at 0.2 µg/kg/min. The initial dose was the same for the Dexmedetomidine group: 1 µg/kg infused in 15 minutes and continued with a maintenance infusion of 0.5 µg/kg/h. Pain and sedation scores and the requirement for analgesic therapy were recorded at 30, 60, 120, and 360 minutes after entering the recovery area [39].

Importantly, baseline characteristics, including age, gender, BMI, prevalence of diabetes mellitus, and hypertension, showed significant differences between the two groups. Notably, Dexmedetomidine was associated with a marked reduction in intraoperative blood pressure and heart rate compared to Remifentanil (p < 0.01), which aligns with its known side effects. Moreover, patients receiving Dexmedetomidine exhibited higher mean sedation scores post-extubation (p < 0.01). Conversely, those in the Remifentanil group had a quicker awakening time (p < 0.001). During the first 30 and 60 minutes of recovery, there was a greater need for analgesic therapy in the Remifentanil group compared to the Dexmedetomidine group (p < 0.05). The mean post-extubation pain scores were also higher in the Remifentanil group (0.4 ± 2.1 vs. 0.3 ± 1.8; p = 0.03). However, after six hours post-extubation, pain scores between the groups converged and were not significantly different (0.4 ± 2.9 vs. 0.8 ± 2.7; p = 0.2). Feedback from surgeons indicated a higher satisfaction rate with the outcomes observed in the Dexmedetomidine group compared to the Remifentanil group (100% vs. 78.6%; p = 0.01) [39].

Beloeil et al. (2021) conducted a rigorous investigator-initiated, prospective, multicenter, parallel-group, single-blind, randomized, and controlled trial involving 314 patients aged 18 years or older who were scheduled for primary or intermediate non-cardiac surgery. The patients were randomly assigned into two groups, each comprising 157 individuals: one receiving Remifentanil and the other Dexmedetomidine. All patients were administered Propofol (1.5-2 mg/kg) followed by Desflurane, along with intravenous Lidocaine (1.5 mg/kg bolus plus 1.5 mg/kg/h), Ketamine (0.5 mg/kg bolus plus 0.25 mg/kg/h), neuromuscular blockade, and Dexamethasone (8 mg) bolus. The Remifentanil group received intravenous Remifentanil via effect-site target-controlled infusion mode (3 to 5 nanograms/mL, equivalent to 0.1 to 0.25 µg/kg/min), while the Dexmedetomidine group was given Dexmedetomidine at an infusion rate of 0.4-1.4 µg/kg/h [40].

The primary outcome of the study focused on a composite of postoperative opioid-related adverse events occurring within the first 48 hours post-extubation, assessed every six hours. These included postoperative hypoxemia (defined as SpO_2_ levels below 95% requiring oxygen supplementation), postoperative ileus (characterized by the absence of flatus or stools), and postoperative cognitive dysfunction evaluated through the Confusion Assessment Method. Secondary outcomes measured included episodes of postoperative pain, opioid consumption within 48 hours after extubation, time taken to achieve an Aldrete score greater than 9 after stopping either Remifentanil or Dexmedetomidine, extubation times, unplanned ICU admissions, PONV, the need for rescue antiemetics, and the duration of hospital stay [40].

The incidence of hypoxemia was observed in 110 out of 152 patients in the Dexmedetomidine group compared to 94 out of 155 patients in the remifentanil group (p = 0.030). However, the mean duration of hypoxemia did not significantly differ between the groups (343 ± 575 minutes in Dexmedetomidine vs. 406 ± 606 minutes in Remifentanil, p = 0.370). Postoperative ileus was present in 33 out of 149 patients in the Dexmedetomidine group and 28 out of 151 in the Remifentanil cohort (p = 0.473). Cognitive function assessments yielded similar results across both groups, with 2 out of 141 patients in the Dexmedetomidine group experiencing cognitive dysfunction compared to none in the Remifentanil group (p = 0.498) [40].

The study was terminated prematurely due to five documented cases of severe bradycardia in patients receiving Dexmedetomidine. Notably, morphine consumption within the first 48 hours post-extubation was significantly lower in the Dexmedetomidine group (6 mg) compared to the Remifentanil group (11 mg, p = 0.002). Additionally, the duration of stay in PACU was longer for the Dexmedetomidine group at 2.28 ± 2.11 hours compared to 1.53 ± 1.47 hours in the Remifentanil group (p = 0.10). There was a tendency toward fewer documented instances of PONV within the first 48 hours in the Dexmedetomidine group (37 instances) versus the Remifentanil group (58 cases, p = 0.10), while the overall duration of hospital stay was similar between the two groups, with the Remifentanil group averaging 5.1 ± 4.5 hours and the Dexmedetomidine group 5.4 ± 5.6 hours (p = 0.664) [40].

Table 3 presents the key findings from studies that directly compared Dexmedetomidine and Remifentanil in terms of their efficacy in post-surgical pain management and their effectiveness in reducing reliance on opioid-based and other analgesic medications.

**Table 3:** Key studies directly comparing Dexmedetomidine with Remifentanil The table presents the characteristics and key findings of studies that directly compared the safety and efficacy of Dexmedetomidine and Remifentanil, examining their effectiveness in providing post-surgical pain relief and their ability to reduce the use of opioid-based and other analgesics.

Authors	Study design	Level of evidence	Sample size	Type of surgery	Key findings	Was Dexmedetomidine comparable to or more effective than Remifentanil in providing post-surgical pain relief and/or reducing the need for opioid-based or other analgesics?
Hwang et al. (2015) [31]	Randomized controlled study	Level A, 1b	37	Posterior lumbar interbody fusion	Dexmedetomidine was found to be effective in reducing postoperative pain and minimizing the necessity for rescue analgesics when compared to Remifentanil. However, it is essential to note that recovery was observed to be slower with the use of Dexmedetomidine.	Yes
Kim et al. (2021) [32]	Prospective randomized double-blinded study	Level A, 1b	61	Laryngeal microsurgery	Dexmedetomidine significantly decreased postoperative pain and the consumption of opioids compared to Remifentanil. However, it was associated with longer recovery times and delayed eye-opening.	Yes
Koo et al. (2023) [33]	Prospective randomized controlled trial	Level A, 1b	96	Gynecological laparoscopy	Dexmedetomidine effectively lowered postoperative pain by 30 minutes and reduced the need for analgesics in the recovery room. Both groups had comparable extubation and eye-opening times, and pain scores were not significantly different at 24 hours post-surgery.	Yes
Hamed et al. (2019) [34]	Prospective randomized controlled trial	Level A, 1b	132	Laparoscopic sleeve gastrectomy	Dexmedetomidine decreased sevoflurane usage, postoperative pain, and the need for opioids for up to 24 hours compared to Remifentanil.	Yes
Goettel et al. (2016) [35]	Prospective randomized controlled trial	Level A, 1b	50	Awake craniotomy	Dexmedetomidine effectively lowered pain scores during skin incision and brain mapping procedures. It also significantly reduced mean arterial pressure and heart rate compared to the control group while maintaining typical respiratory rates. These findings indicated that Dexmedetomidine was an excellent alternative for pain management, leading to reduced pain and decreased reliance on opioids.	Yes
Bakan et al. (2015) [36]	Prospective randomized double-blinded study	Level A, 1b	80	Laparoscopic cholecystectomy	The Dexmedetomidine group had statistically significantly less cumulative postoperative fentanyl use in the first two hours. The opioid-based group had more hypotension treated with ephedrine. These findings suggest that Dexmedetomidine reduced postoperative opioid use and associated side effects despite a small sample size.	Yes
Pan et al. (2019) [37]	Randomized controlled trial	Level A, 1b	94	Radiofrequency ablation of hepatocellular carcinoma	Dexmedetomidine was associated with notably lower heart rates throughout surgery and recovery, as well as a decrease in sevoflurane consumption. Emergence and extubation times were comparable, with no significant occurrences of bradycardia or hypotension.	Yes
Janatmakan et al. (2021) [38]	Double-blind randomized clinical trial	Level A, 1b	60	Lumbar discectomy	Dexmedetomidine significantly reduced mean pain intensity compared to the Remifentanil group. Additionally, while both groups had no differences in heart rate or blood pressure before, during, or after surgery, the Dexmedetomidine group exhibited notably lower mean arterial pressure and heart rate.	Yes
Rahimzadeh et al. (2015) [39]	Double-blind randomized clinical trial	Level A, 1b	60	Posterior spinal fusion surgery	Dexmedetomidine effectively lowered intraoperative blood pressure and heart rate, improved sedation scores after extubation, and reduced pain and the need for analgesics in the first 60 minutes post-recovery compared to Remifentanil. However, Remifentanil facilitated faster awakening times. Six hours after extubation, pain scores were comparable between the two groups.	Yes
Beloeil et al. (2021) [40]	Prospective single-blind, randomized controlled trial	Level A, 1b	314	Major or intermediate non-cardiac surgery	In the first 48 hours following surgery, average morphine consumption was lower in the Dexmedetomidine group compared to the Remifentanil group.	Yes

Discussion

The emergence of OIH and opioid dependency presents significant challenges in postoperative pain management, underscoring the urgent need for alternatives to traditional opioid-based analgesics. This systematic review evaluates the comparative efficacy of Dexmedetomidine and Remifentanil in reducing postoperative pain and opioid consumption. While both agents have demonstrated efficacy in clinical settings, their mechanisms, safety profiles, and long-term effects reveal crucial insights for improving patient outcomes.

Remifentanil, a μ-opioid receptor agonist, is a widely used short-acting opioid in anesthesia. It provides rapid onset and offset of analgesia, making it suitable for short surgical procedures. However, its use is not without limitations. One of the significant concerns with Remifentanil is its association with OIH, a condition where prolonged opioid exposure paradoxically increases pain sensitivity postoperatively. Numerous studies have observed this phenomenon, including those by Hwang et al. (2015) and Koo et al. (2023), where patients administered Remifentanil required greater rescue analgesia. The desensitization of opioid receptors following repeated exposure is believed to exacerbate postoperative pain and increase the demand for opioids. Consequently, this leads to prolonged hospital stays, higher healthcare costs, and an increased risk of chronic opioid use [31,33].

On the other hand, Dexmedetomidine, an α2-adrenergic receptor agonist, offers a non-opioid mechanism for managing postoperative pain. It exerts its analgesic effects by inhibiting the release of norepinephrine in the CNS, effectively modulating pain pathways without stimulating opioid receptors, which is particularly advantageous as it reduces the risk of OIH and opioid dependency. Additionally, Dexmedetomidine is associated with minimal respiratory depression, making it a safer alternative for opioid-free anesthesia. The studies consistently show that Dexmedetomidine leads to reduced postoperative pain and a lower requirement for opioid-based rescue analgesia, as evidenced by multiple randomized controlled trials [34-40].

*Efficacy in Postoperative Pain Managemen*t

A critical aspect of postoperative pain management is the ability to control pain effectively while minimizing opioid use. The studies reviewed reveal that Dexmedetomidine consistently outperforms Remifentanil in reducing postoperative pain. For instance, Kim et al. (2021) found that patients who received Dexmedetomidine had significantly lower postoperative pain scores and required fewer opioids compared to the Remifentanil group following laryngeal microsurgery [32]. Similarly, Koo et al. (2023) observed that Dexmedetomidine resulted in significantly lower pain scores in the recovery room than Remifentanil in gynecological laparoscopy patients [33]. Furthermore, Hwang et al. (2015) demonstrated that Dexmedetomidine reduced the need for rescue analgesics in spinal surgery patients, thereby reducing overall opioid consumption [31].

Despite the superiority of Dexmedetomidine in reducing pain and opioid consumption, it is crucial to note the trade-offs in recovery dynamics. Studies, such as those by Kim et al. (2021) and Goettel et al. (2016), indicate that Dexmedetomidine is associated with slower recovery times, particularly regarding delayed eye-opening and extubation. These delays concern specific patient populations, particularly those requiring quick postoperative mobilization. However, the benefits of reduced opioid use and associated risks of OIH may outweigh the slower recovery for many patients, especially those at risk of opioid misuse or dependence [32,35].

Hemodynamic Considerations

In addition to pain relief, hemodynamic stability is crucial in choosing anesthetic agents. Remifentanil and Dexmedetomidine have been shown to influence hemodynamics, but their effects differ. Remifentanil is known for its rapid onset and offset of action, which can lead to significant heart rate and blood pressure fluctuations, particularly during induction and emergence from anesthesia. In contrast, Dexmedetomidine induces more stable hemodynamics, with a slower and more controlled heart rate and blood pressure reduction [35-40]. This has been confirmed, including those by Rahimzadeh et al. (2015) and Kim et al. (2021), where Dexmedetomidine demonstrated better hemodynamic stability than Remifentanil. While Dexmedetomidine's cardiovascular effects include bradycardia and hypotension, these are generally manageable and less severe than the fluctuations associated with Remifentanil, particularly in the early postoperative period [32,39].

Safety and Side Effects

The safety profile of both agents has been extensively studied. Dexmedetomidine's primary side effects include bradycardia, hypotension, and sedation. These effects are generally dose-dependent and can be reduced with careful titration of the infusion rate. In contrast, Remifentanil's side effects are predominantly related to its opioid nature, including the risk of OIH and respiratory depression. While both drugs are associated with mild adverse effects, Dexmedetomidine's non-opioid mechanism offers a significant advantage in avoiding the long-term risks associated with opioid use, such as dependence and addiction.

It is essential to consider the results of Beloeil et al. (2021), which noted an increased incidence of bradycardia in patients receiving Dexmedetomidine compared to Remifentanil. While these findings suggest that bradycardia is an adverse effect, it did not result in significant clinical complications. Nevertheless, careful monitoring of cardiovascular parameters is essential when using Dexmedetomidine, particularly in high-risk patients [40].

Limitations and Future Directions

Despite the promising findings from the studies reviewed, notable limitations warrant consideration. First, most of the studies included in this review had relatively small sample sizes, which may limit the generalizability of the results. More extensive trials are necessary to confirm the robust benefits of Dexmedetomidine over Remifentanil, especially in diverse patient populations.

Second, the studies reviewed largely focused on specific surgical procedures, such as spinal surgery, gynecological laparoscopy, and laryngeal microsurgery. It is unclear whether the findings would be consistent with macrosurgery, particularly those with different pain profiles or complexities. Future studies should explore the efficacy of Dexmedetomidine in a broader range of surgical settings, including high-risk procedures and those with significant comorbidities.

Additionally, the long-term effects of Dexmedetomidine use on postoperative recovery, particularly in terms of cognitive function and quality of life, remain underexplored. While several studies have demonstrated a reduction in opioid consumption, there is limited data on how these effects impact long-term outcomes such as chronic pain, opioid dependency, or overall recovery trajectories.

Finally, the optimal dosing regimen for Dexmedetomidine remains a topic for further investigation. While many studies utilized continuous infusions, the most effective dose and duration for minimizing both pain and opioid consumption while minimizing adverse effects (e.g., bradycardia) has yet to be established. Comparative studies with other non-opioid agents, including regional anesthetics, would also be beneficial in assessing the relative efficacy and safety of Dexmedetomidine as part of multimodal analgesia strategies.

## Conclusions

This systematic review highlights the promising role of Dexmedetomidine as an alternative to Remifentanil for postoperative pain management. Dexmedetomidine offers superior analgesic efficacy, significantly reducing opioid consumption and mitigating the risk of OIH, a concern that is particularly relevant in the context of the opioid crisis. While its slower recovery profile and hemodynamic effects may present challenges in certain settings, the overall benefits - especially in terms of reducing opioid-related complications - make Dexmedetomidine a valuable tool in the perioperative anesthesiologist's armamentarium. Further large-scale studies with more diverse patient populations are needed to fully evaluate the long-term benefits and risks of Dexmedetomidine compared to Remifentanil.
